# Evaluation of Automated Molecular Testing Rollout for Tuberculosis Diagnosis Using Routinely Collected Surveillance Data — Uganda, 2012–2015

**DOI:** 10.15585/mmwr.mm6612a6

**Published:** 2017-03-31

**Authors:** Colleen Scott, Simon Walusimbi, Bruce Kirenga, Moses Joloba, Muttamba Winters, Nyombi Abdunoor, Rommel Bain, Heather Alexander, Tom Shinnick, Sean Toney, Rosemary Odeke, Christine Mwangi, Estella Birabwa, Seyoum Dejene, Frank Mugabe, Mukadi YaDiul, J. Sean Cavanaugh

**Affiliations:** ^1^Epidemic Intelligence Service, CDC; ^2^Division of Global HIV and TB, CDC; ^3^Lung Institute Makerere University, College of Health Sciences, Kampala, Uganda; ^4^TB/Leprosy Control Program, Ministry of Health, Republic of Uganda, Kampala Uganda; ^5^Division of Global HIV and TB, CDC, Uganda; ^6^U.S. Agency for International Development, Uganda; ^7^U.S. Agency for International Development, Washington DC.

In 2012, Uganda introduced the use of GeneXpert MTB/RIF (Cepheid, Sunnyvale CA), a sensitive, automated, real-time polymerase chain reaction–based platform for tuberculosis (TB) diagnosis, for programmatic use among children, adults with presumptive human immunodeficiency virus (HIV)-associated TB, and symptomatic persons at risk for rifampicin (RIF)-resistant TB. The effect of using the platform’s Xpert MTB/RIF assay on TB care and control was assessed using routinely collected programmatic data; in addition, a retrospective review of district quarterly summaries using abstracted TB register data from purposively selected facilities in the capital city of Kampala was conducted. Case notification rates were calculated and nonparametric statistical methods were used for analysis. No statistically significant differences were observed in case notification rates before and after the Xpert MTB/RIF assay became available, although four of 10 districts demonstrated a statistically significant difference in bacteriologically confirmed TB. Once the GeneXpert MTB/RIF platform is established and refined, a more comprehensive evaluation should be conducted.

The Xpert MTB/RIF assay detects genetic sequences of *Mycobacterium tuberculosis* complex as well as mutations associated with resistance to RIF and provides results in 2 hours. The test is much more sensitive than the conventional diagnostic test (sputum smear microscopy), with a pooled sensitivity among persons living with HIV infection of 80% (*1*). The World Health Organization recommends use of the Xpert MTB/RIF assay as the initial diagnostic test in adults and children with presumptive HIV-associated TB or multidrug resistant TB (*2*). It is hoped that the use of a more sensitive diagnostic test will increase case detection and notification; however, an evaluation of the Xpert MTB/RIF assay in Nepal found that use of Xpert MTB/RIF testing was associated with an increase in the proportion of TB diagnoses that were bacteriologically confirmed, but had little impact on overall rate of diagnoses or patient care, which might be the case in locations where clinical diagnosis and empiric TB treatment are common (*3*).

In Uganda, the HIV prevalence in adults is >7% (*4*), and the Xpert MTB/RIF assay is used as the initial diagnostic test for all persons living with HIV, children, and persons at risk for RIF-resistant TB who have any of the principal signs or symptoms of TB (cough, weight loss, night sweats, or fever). As of February 2016, there were 111 GeneXpert instruments installed in 76 (68%) of 111 districts throughout Uganda.

Two retrospective data reviews were conducted. The first was a review of district quarterly reports from 2012 to 2015 submitted to the National Tuberculosis and Leprosy Program; regional case notification rates before and after availability of GeneXpert MTB/RIF testing were compared. Ten districts that had data reported and available for multiple quarters before and after the installation of a GeneXpert instrument were selected, and deidentified data from multiple calendar-year quarters before and after GeneXpert instruments were installed were abstracted. Case notification rates were calculated using the Uganda National Population and Housing Census 2014 (*5*). For the second review, line-listed data (including longitudinal data such as treatment outcomes) were abstracted on all patients registering for TB therapy during 2012–2015 at a convenience sample of six facilities in Kampala, which were selected based on size, ease of access, and completeness of records. At five facilities, data were collected from patients registered during one quarter before and two quarters after the availability of Xpert MTB/RIF assays; at four of those facilities, data were collected over a 24-month period, and at the fifth, data were collected over an 18-month period. Because of high patient volume at the sixth facility (Mulago National Referral Hospital), data were collected from patients registered during the first month of the quarter immediately before introduction of Xpert MTB/RIF testing, and the first month of each of the two quarters immediately after introduction of Xpert MTB/RIF testing.

The Wilcoxon rank sum test was used to test for differences in case notification rates between districts before and after Xpert MTB/RIF testing initiation, and differences were considered statistically significant if p<0.05. Because of small sample sizes and uncertainty about the population from which the samples were drawn, nonparametric bootstrap sampling was used to construct confidence intervals for the difference in facility diagnoses before and after installation of GeneXpert instruments. Bootstrap sampling was also used to evaluate treatment outcomes reported by health facilities, specifically evaluating the differences between facilities in the proportion of patients with TB in three mutually exclusive categories: 1) completed TB treatment, 2) stopped TB treatment without completing, and 3) continuing TB treatment at the time of data collection. A total of 100,000 bootstrap samples were used to approximate the true sampling distribution for each model.

Forty quarterly report summaries from the 10 selected districts were abstracted. Although no statistically significant differences in case notification rates before and after Xpert MTB/RIF testing initiation were identified, statistically significant increases in the percentage of bacteriologically confirmed TB cases were found in four districts (Table 1).

**TABLE 1:** Median case notification rates and percentage of cases bacteriologically confirmed before and after Xpert MTB/RIF availability, by selected district (N = 10) — Uganda, 2012–2015*

Region	District	No. quarters^†^ before Xpert MTB/RIF	No. quarters^†^ after Xpert MTB/RIF	Median case notifications per 100,000 population			Median percentage bacteriologically confirmed		
				Before Xpert MTB/RIF	After Xpert MTB/RIF	p value	Before Xpert MTB/RIF	After Xpert MTB/RIF	p value
Northern	Arua	7	2	23	23	0.58	52	62	0.09
Northern	Kitgum	5	3	47	39	0.80	48	67	0.02**^§^**
Western	Kabale	5	4	19	21	0.50	64	71	0.06
Western	Kabarole	5	4	34	31	0.87	54	68	0.14
Western	Kisoro	8	3	26	16	0.97	46	55	0.09
Western	Ntungamo	7	3	20	19	0.96	74	89	0.13
Eastern	Mbale	6	4	38	38	0.67	58	73	0.02**^§^**
Eastern	Tororo	6	6	31	27	0.99	50	55	0.03**^§^**
Central	Mpigi	6	6	26	33	0.17	77	68	0.99
Central	Rakai	7	5	24	29	0.17	67	77	0.01**^§^**

* Based on Wilcoxon rank sum test.

^†^ 3-month calendar period.

^§^ Statistically significant (p≤0.05).

A total of 1,650 patient records were abstracted from the six Kampala facility treatment registers. Records from one (Kiseny Health Center IV) indicated a statistically significant increase in the proportion of TB cases that were bacteriologically confirmed after availability of Xpert MTB/RIF testing (Table 2). This health facility also had a statistically significant increase in the proportion of patients who completed TB treatment after Xpert MTB/RIF testing initiation and a decrease in the proportion who stopped treatment before completion. In a second facility (Nsambya Hospital), records indicated a statistically significant decrease in the proportion of patients completing treatment and an increase in the proportion of TB cases continuing in TB treatment (Table 2).

**TABLE 2:** Difference in proportion of bacteriologically confirmed* TB cases before and after Xpert MTB/RIF installation, and Bootstrap mean difference estimates and 95% CIs for treatment outcomes, by health facility (N = 6) — Kampala, Uganda, 2012 – 2015

Characteristics	Health facility					
	Alive Medical Services	Kisenyi Health Center IV	Kisugu	Mengo	Mulago Ward 5 and 6	Nsambya Hospital
Difference in proportion of bacteriologically confirmed* TB cases %, (95% CI)	8.3 (−3.1 to 29.8)	30.8 (21.3 to 40.2)^†^	14.9 (−3.8 to 33.3)	−10.1 (−26.3 to 6.3)	−1.7 (−12.6 to 9.3)	5.1 (−14.0 to 24.2)
**Bootstrap mean difference estimates (95% CI)^§^ for TB treatment outcomes**						
TB treatment completed	−0.119 (−0.357 to 0.119)	0.184 (0.059 to 0.307)^†^	−0.153 (−0.364 to 0.056)	−0.012 (−0.130 to 0.097)	−0.064 (−0.169 to 0.040)	−0.728 (−0.839 to −0.598)
Stopped TB treatment before completion	0.000 (−0.214 to 0.214)	−0.179 (-0.292 to 0.063)^†^	0.071 (−0.105 to 0.249)	0.040 (−0.056 to 0.149)	0.030 (−0.063 to 0.125)	−0.018 (−0.134 to 0.098)
Continuing TB treatment	0.119 (−0.048 to 0.298)	−0.006 (−0.081 to 0.074)	0.082 (−0.051 to 0.233)	−0.028 (−0.079 to 0.031)	0.034 (−0.031 to 0.102)	0.746 (0.608 to 0.866)^†^

**Abbreviations:** CI = confidence intervals; TB = tuberculosis.

* Bacteriologically confirmed TB includes cases diagnosed using either GenXpert or culture.

^†^ Statistically significant (p≤0.05).

^§^ Bootstrap percentile CIs using 100,000 samples per model.

## Discussion

This early impact evaluation of the rollout of Xpert MTB/RIF testing did not demonstrate an apparent increase in overall TB case notification rates after testing became available in Uganda, although the proportion of bacteriologically confirmed TB cases increased in a few selected districts. Both findings validate previous reports (*3*,*6*,*7*).

Overall, there were no observable differences in treatment outcomes before and after Xpert MTB/RIF testing availability in reviewed health facilities in Kampala, although there was an apparent increase in TB treatment completion in one facility (Kisenyi). Time from specimen collection to treatment initiation (time to treatment), which elsewhere has been reduced by Xpert MTB/RIF test availability and use (*8*,*9*), was not evaluated in this analysis. Reducing time to treatment would be expected to reduce transmission, and could have an epidemiologic impact; moreover, reducing time to treatment might improve outcomes for the sickest patients and patients with multidrug resistant TB.

The lack of effect on TB case notification rates likely reflects the overall low usage rates, given that Xpert MTB/RIF testing was available only to a minority of patients with presumptive TB disease and might have been underused even in the target populations, and also corroborates findings from a previously reported facility-level review (*10*). It is also possible that Xpert MTB/RIF testing might be replacing clinically diagnosed cases, which represented a large proportion of TB cases before Xpert MTB/RIF testing became available, with biologically confirmed cases, as has been suggested in other similar evaluations (*6*). In addition, this might be partially explained by overestimation of the test’s sensitivity by clinical staff members. If staff members assume a negative test is definitive, leaving them reluctant to make a clinical diagnosis, then Xpert MTB/RIF testing might have the paradoxical effect of decreasing the likelihood of diagnosing those with bacillary burdens below the level of detection. This possibility merits investigation with focused research; if found to be true, additional training on the sensitivity of the Xpert MTB/RIF assay and the importance of complete clinical appraisal of persons with suspected TB might lead to improved case detection. 

The findings in this report are subject to at least five limitations. First, the sampling and the geographic focus of the facility data limit definitive and generalizable conclusions. Second, bootstrapping methods assume the original sample represents the population from which the sample was drawn; as such, the facility-level findings are generalizable only to those facilities. Third, because the study was conducted shortly after Xpert MTB/RIF testing became programmatically available (i.e., during the first 6 months of introduction), limited experience might have resulted in suboptimal usage of the test, misinterpretation of test results, and unreliable data recording. Fourth, because routine programmatic data were used for district-level analyses, it is possible some data were incomplete or erroneous. Finally, data on severity of patient illness, such as clinical stage of HIV infection or CD4 cell count, were not collected, and the number of RIF-resistant TB cases in the sample was very few, precluding assessment of the impact of the Xpert MTB/RIF assay on treatment outcomes in specific subpopulations.

The effect of Xpert MTB/RIF testing on TB case notification has not yet been fully realized in Uganda. Findings from this evaluation will help direct operations research, such as a review of the algorithm for TB diagnosis, as well as programmatic interventions, such as training health care workers on using Xpert MTB/RIF tests and interpreting results. Once the GeneXpert platform is fully established and made more widely available, the national program could consider conducting a reevaluation of the impact of the Xpert MTB/RIF assay and a review of the diagnostic algorithm for TB in Uganda to validate and expand these findings. Additional studies might include a longitudinal study to conduct a more targeted evaluation of the overall introduction of Xpert MTB/RIF testing and the effects on clinical diagnoses, the impact of Xpert MTB/RIF testing on the sickest patients and those with RIF-resistant disease, and an assessment of feasibility and effect of expanding the Xpert MTB/RIF testing algorithm.

**Summary**

**What is already known about this topic?**

The World Health Organization recommends use of the Xpert MTB/RIF assay as the initial diagnostic test in adults and children with presumptive HIV-associated TB or multidrug-resistant TB. Currently, data on the effect of the Xpert MTB/RIF assay on case notification or TB treatment outcomes are limited. Published studies indicate the Xpert MTB/RIF assay might improve the proportion of TB diagnoses that are bacteriologically confirmed, but appears to have little effect on overall rate of diagnoses or patient care, especially in locations where clinical diagnosis and empiric TB treatment are high.

**What is added by this report?**

This early impact evaluation of the Xpert MTB/RIF rollout demonstrated no apparent increase in overall TB case notification rates after testing became available in Uganda. However, within a few selected districts the proportion of bacteriologically confirmed TB cases did increase after testing became available. These two findings validate previous reports.

**What are the implications for public health practice?**

The impact of Xpert MTB/RIF testing on TB case notification has not yet been fully realized in Uganda. Findings from this evaluation will help direct operations research, such as a review of the diagnostic algorithm for TB, as well as programmatic interventions, such as training health care workers on Xpert MTB/RIF usage and results interpretation.
